# Urban aliens and threatened near-naturals: Land-cover affects the species richness of alien- and threatened species in an urban-rural setting

**DOI:** 10.1038/s41598-020-65459-2

**Published:** 2020-05-22

**Authors:** Tanja K. Petersen, James D. M. Speed, Vidar Grøtan, Gunnar Austrheim

**Affiliations:** 1 Department of Natural History, NTNU University Museum, Norwegian University of Science and Technology (NTNU), Erling Skakkes gt 47b, Trondheim, Norway; 2 Centre for Biodiversity Dynamics, Department of Biology, NTNU, NO-7491, Trondheim, Norway

**Keywords:** Biodiversity, Conservation biology, Ecological modelling, Invasive species, Urban ecology

## Abstract

Urbanisation has strong effects on biodiversity patterns, but impacts vary among species groups and across spatial scales. From a local biodiversity management perspective, a more general understanding of species richness across taxonomic groups is required. This study aims to investigate how fine-scale land-cover variables influence species richness patterns of locally threatened and alien species. The study was performed in Trondheim, Norway, covering a steep urbanisation gradient. Spatially correlated Generalised Linear Mixed Effects Models predicting the number of all-, threatened-and alien species by taxon, habitat, habitat heterogeneity and mean aspect within 500 m×500 m grid cells were constructed. The habitat categories were based on detailed land-cover maps. The highest number of threatened species was found in habitats relatively less affected by humans, whereas the number of alien species were only dependent on taxonomic group and spatial correlation. It is shown that land-cover variables within an administrative border can be used to make predictions on species richness within overarching species groups. Recommendations to biodiversity management agencies are to ensure protection of natural habitats to favour locally threatened species, and closely monitor urban areas to mitigate the introduction and spread of alien species.

## Introduction

The majority of the world’s population now live in cities, and urbanisation is predicted to increase further in the future^1,2^. Cities are frequently located in biodiversity hotspots, and increases in urban areas inevitably will happen at the cost of other habitats important for biodiversity^3–6^. This calls for studies detailing how to manage biodiversity efficiently and properly in urban areas.

Various effects of urbanisation on biodiversity have been suggested and reported, depending on the exact variables in question, and the trends differ among taxa^5,7,8^. For example, urbanisation can be a homogenising force on multiple spatial scales, impoverishing the local native species pool^7,9,10^, while supplying alien species^11,12^. Thus, alpha diversity might increase, despite larger-scale beta diversity decrease^13,14^. The positive correlation between plant species richness and urbanisation can be caused by high habitat heterogeneity in urban areas, due to patches of remnant (semi-)natural habitats, allowing species with different requirements to persist^15^. Other reasons can be the introduction of alien plant species, e.g. for ornamental purposes, and a natural high productivity independent of human settlement^7,11,16^.

Cadotte *et al*. (2017)^17^ reported that alien species richness generally increases with urbanisation. In contrast, other studies have linked urban areas with relatively high numbers of native and/or threatened species (see e.g. Kühn and Klotz (2006)^14^, Kowarik (2011)^5^ and references, and Ives *et al*. (2016)^18^).

Studies of biodiversity in relation to land use, urbanisation and human disturbance have been carried out on vastly different spatial scales (meters to>1000 km) (e.g. Blair (1996)^13^, Kühn and Klotz (2006)^14^, Pautasso (2007)^19^, Ahrne *et al*. (2009)^20^ and Bertolino *et al*. (2015)^21^). Studies on a fine spatial scale, including a broad urbanisation gradient (ranging from industrialised to natural areas) are largely lacking however (but see Turrini and Knop (2015)^22^ and Concepción *et al*. (2016)^23^)^20^. If the results of biodiversity research are to be used by local management, it is crucial that these results are obtained and delivered on a relatively small spatial scale, appropriate for potential management intervention. Hence, this study is performed on a spatial scale in close accordance with the spatial scale of urban planning and management.

For conservation purposes, focus is frequently placed on restricted groups, such as protection of native, threatened species or mitigation of alien species (see e.g. the Norwegian ‘Natural diversity’ law^24^, the EU Habitats Directive^25^, and the EU Regulation on Invasive Alien Species^26^). In particular, the similarities and differences in variables determining their distributions are of interest, e.g. alien species being favoured by urbanisation, whereas native species can be threatened by such^27,28^. Knowledge of how broad land-cover variables affect the distribution and richness of these groups could help guide decisions on city development and biodiversity management on municipality level. Threatened and alien species have rarely been investigated simultaneously (but see Deutschewitz *et al*. (2003)^29^, Kühn *et al*. (2004)^16^ and Matthies *et al*. (2017)^30^.

The aim of this study is to investigate which general, fine-scale land-cover variables influence species richness patterns of all species, as well as specifically rare and non-native species. As a case study system, we use a northern European municipality with a strong urban to rural gradient. We predict that:

*Urban areas* are predicted to have higher levels of alien species richness compared to non-urban areas, as cities function as introduction sites for (plant) species associated with gardens. Similarly, key pathways for introduction of alien species are through trade and traffic, which are more prevalent in urban areas than outside^5,10,17,31,32^.

Urban areas are predicted to have relatively low levels of threatened species richness due to the high level of disturbance in urban areas^7,8^. However, naturally high levels of biodiversity or suitable microhabitats within the urban matrix can potentially lead to the opposite pattern^5,16,18^. Urban areas are viewed here as areas dominated by build-up area and immediately surrounding areas.

*Forests* are predicted to have high levels of both threatened- and alien species richness, as approx. 48% of the Norwegian Red-listed species are generally affiliated with forests, while several alien tree species have been planted for forestry purposes throughout Norway^33–35^ The associations between species richness and forest cover might depend on more fine-scale forest composition and structure, out of scope of this study.

*Coastal areas* are ecotones, and are thus expected to host a high number of species^36^.

*Open areas with sparse vegetation* (or otherwise disturbed habitat) are predicted to have high levels of alien species richness, as these are able to exploit disturbed habitat^5,17,27^; e.g. alien plant species with a ruderal life strategy^37^.

*Habitat heterogeneity* affect the richness of both groups positively, as more diverse habitat within an area provide resources for different requirements^4,29,30,36,38–41^.

*Topography:* North-facing slopes are expected to be negatively correlated with overall species richness, as plants are negatively affected by a lack of light and lower temperatures^42^.

## Materials and Methods

### Study area

**Figure 1:**
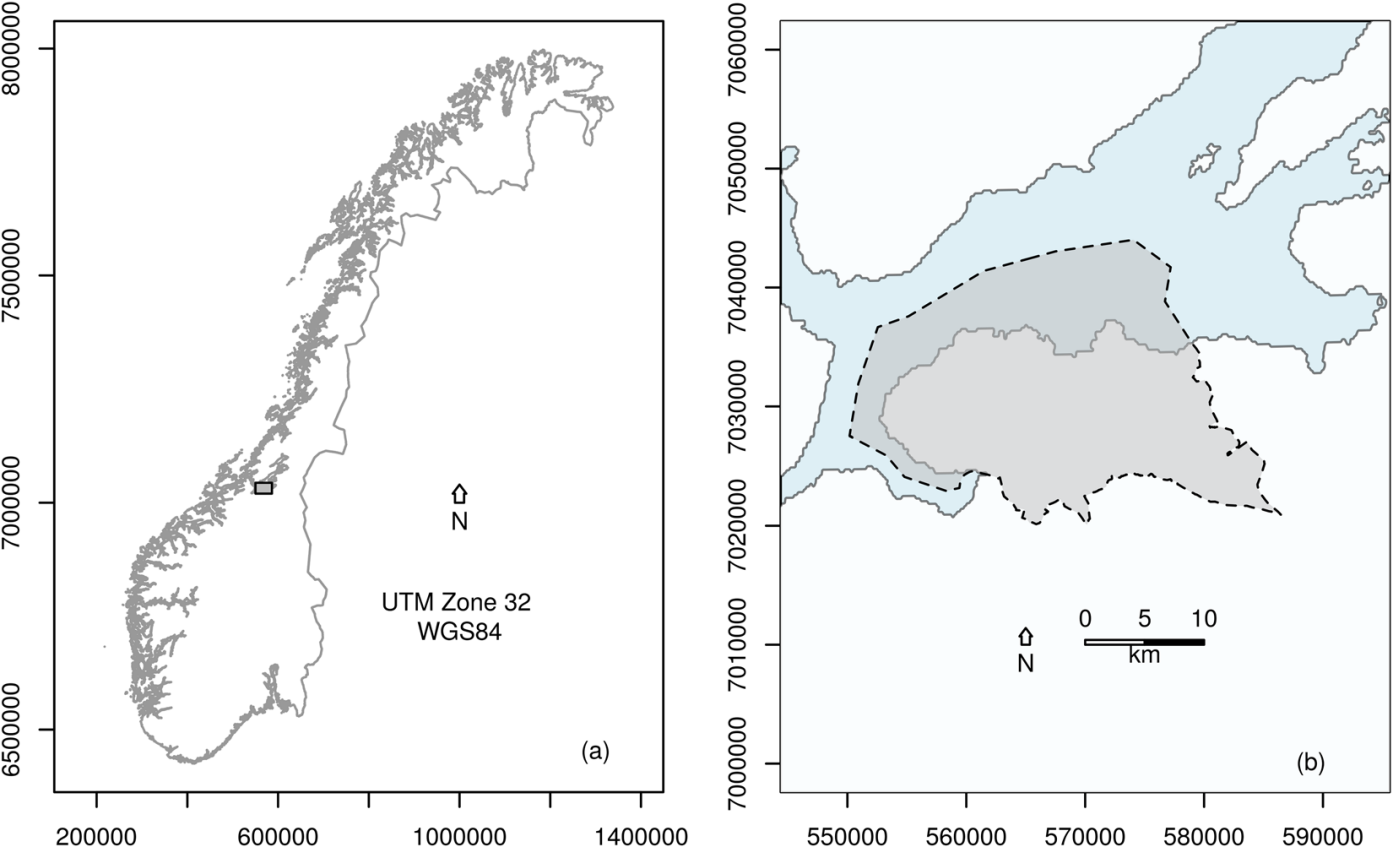
Location of study system. (**a**) Map of Norway, position of Trondheim Municipality indicated with a grey box, (**b**) Trondheim Municipality, municipality border indicated with dashed line and grey colour. The figure was made in R, version 3.6.1^55^.

The study was carried out within Trondheim Municipality (Norway) administrative borders, around 63.42°N, 10.38°E (Fig. 1a,b). It is a southern-boreal^43^, coastal municipality with an area of 342 km^2^, a population of approximately 190,000 people^44^, and annual mean temperature and precipitation are approximately 5 °C and 887 mm^45^. The municipality holds a steep urbanisation gradient; from the city centre and industrial areas, through rural areas including agricultural areas and commercial forests, and to nature reserves and areas managed for biodiversity conservation. The municipality covers highly different nature types, including both coastline, subalpine areas and limnic systems, and thus has a high potential for varied biological communities and high levels of biodiversity^44^. Trondheim municipality is fairly well-sampled with regards to species occurrence records, e.g. due to the presence and activity of the University Museum.

### Data retrieval and data cleaning

#### Land cover data

Land cover was based on the Norwegian AR5 maps (Land Resource map 1:5000) from NIBIO^46^. Shapefiles of the land cover maps were provided by the Trondheim Municipality in April 2018. The AR5 maps are both continually and periodically updated, and provides the most complete data on national land resources^47^. Land cover is categorised based on land cover type, tree cover type, timber productivity and soil condition, giving 66 functionally unique categories within Trondheim municipality (hereafter called “land cover types”) (Supplementary Material 1, Table S.1). The map was overlaid by a 500 m × 500 m grid.

Updates of the AR5 maps are mainly done if the categorical classification of an area changes, and the responsible authorities are notified of this change^46^. Consequently, “unannounced” changes are not reflected in the data. As the land cover data was matched with GBIF records from 2013–2018, changes within this period are not taken into account.

Aspect of the terrain was retrieved from a Digital Terrain Model raster with a resolution of 25 m × 25 m. The circular aspect (unit: degrees) was transformed to a “*northness*”-measure by $Northness=cosin(Aspect(^\circ ))$, hence fitting a scale of −1 to 1 (in this definition: −1 = south-facing, 1 = north-facing). The values were rescaled to a gradient from 0 to 1 to match the scale of other included variables. All flat areas were given NA-values. For each grid cell, northness was calculated as the mean of all raster cells within the overlaid grid cell.

#### GBIF occurrence records

Large amounts of data on species occurrences are available from online databases, such as the Global Biodiversity Information Facility (GBIF)^48^, and the Norwegian Biodiversity Information Centre^49^.

All occurrence records from a bounding box around Trondheim Municipality (the exact municipality border was too detailed to include in the process) were downloaded from GBIF on 06/03-2018^50^ (864,715 records in total, giving 9,117 unique species names; 48,468 records not identified to species level), and subsequently spatially filtered according to the municipality border. The data was additionally filtered according to the following criteria, reducing the number of records to 251,803 across 3,097 unique species names:

1. Records containing a full species name for comparability with the threatened- and alien species lists.
2. Coordinate uncertainty of ≤354 m, (1/2 length of the diagonal of 500 m ×500 m grid cells).
3. Records made between January 1^st^ 2013 and March 6^th^ 2018 to ensure compatibility with the used land cover maps, and a negligible amount of land cover change.

Of these records, 94.9% were within the kingdom Animalia (91.4% of the total data set were birds), 3.7% within Plantae, and 1.3% within Fungi. 0.65‰ (163 records) were from outside these kingdoms (Supplementary material 2, Table S.2). The data set was divided into threatened- and alien species (only including animals, plants and fungi).

The “threatened” status was defined based on one or more assessments of the national Norwegian Red List from 2006, 2010 and 2015, provided by The Norwegian Biodiversity Information Centre. See Supplementary material 3 for detailed description of inclusion details.

The “alien” status was based on the Alien Species List (v. 2012^51^) from The Norwegian Biodiversity Information Centre. Only species alien in mainland Norway were retained (excluding species alien only to Svalbard). All alien species were included, regardless of risk category. Discrepancies in nomenclature between GBIF records and species lists were resolved using the Taxonomic Name Resolver (function “tnrs” from the taxize-package^52^). Only terrestrial and limnic species were included in the data sets. All species classified as marine by The Norwegian Biodiversity Information Centre were manually excluded from the lists (excluding birds; all bird species in the data set were regarded as terrestrial).

32,585 records (121 unique species names) could be categorised as threatened (99.3% animals, 98.9% birds, 0.3% plants and 0.4% fungi), and 3,447 (177 species) as alien (64.6% animals, 63.4% birds, 34.3% plants and 1.1% fungi) (Supplementary material 2, Table S.2). The risk of species mis-identification is considered negligible, as the majority of records are associated with organisations deemed reliable regarding species identification (e.g. the Norwegian Ornithological Society, the Norwegian Botanical Society and the NTNU University Museum herbarium). Furthermore, as individual species are not analysed, it is unlikely that single erroneous records will affect the aggregated species pool.

The number of threatened- and alien species, and the overall species richness, registered within each grid cell was calculated, and divided into five taxonomic groups: birds, non-avian animals, plants, fungi and other taxa. “Other taxa” was excluded from further analyses due to a low amount of data.

### Preparation of variables

#### Land cover variables

To reduce the number of land cover types while avoiding subjectively defining categories, hierarchical cluster analysis was used to identify grid cells with similar composition, creating a limited number of clustered land cover type categories. All grid cells within the administrative border of the Trondheim municipality were used for the cluster analysis, including cells only partially within the municipality border, including only the within-municipality area. Marine grid cells (entirely covered by ocean) were not included, resulting in 1509 grid cells in total.

The cluster analysis was done using the function “hclust” on a dissimilarity matrix based on the AR5 land cover in each grid cell, using “Complete linkage” as the clustering method, and a Bray-Curtis dissimilarity matrix of the individual grid cells (function “vegdist”, package vegan^53^). Cut-off value was set at *height*=0.99 (referring to the height of the cluster-tree, where height=1 indicates no clustering, and height=0 each individual branch (grid cell) being an autonomous cluster), resulting in 17 clusters in total, of which 6 included ≤3 grid cells. The clusters including ≤3 grid cells were mainly found on the municipality border. These were excluded from further analysis. Each cluster will hereafter be referred to as a “habitat”.

**Figure 2:**
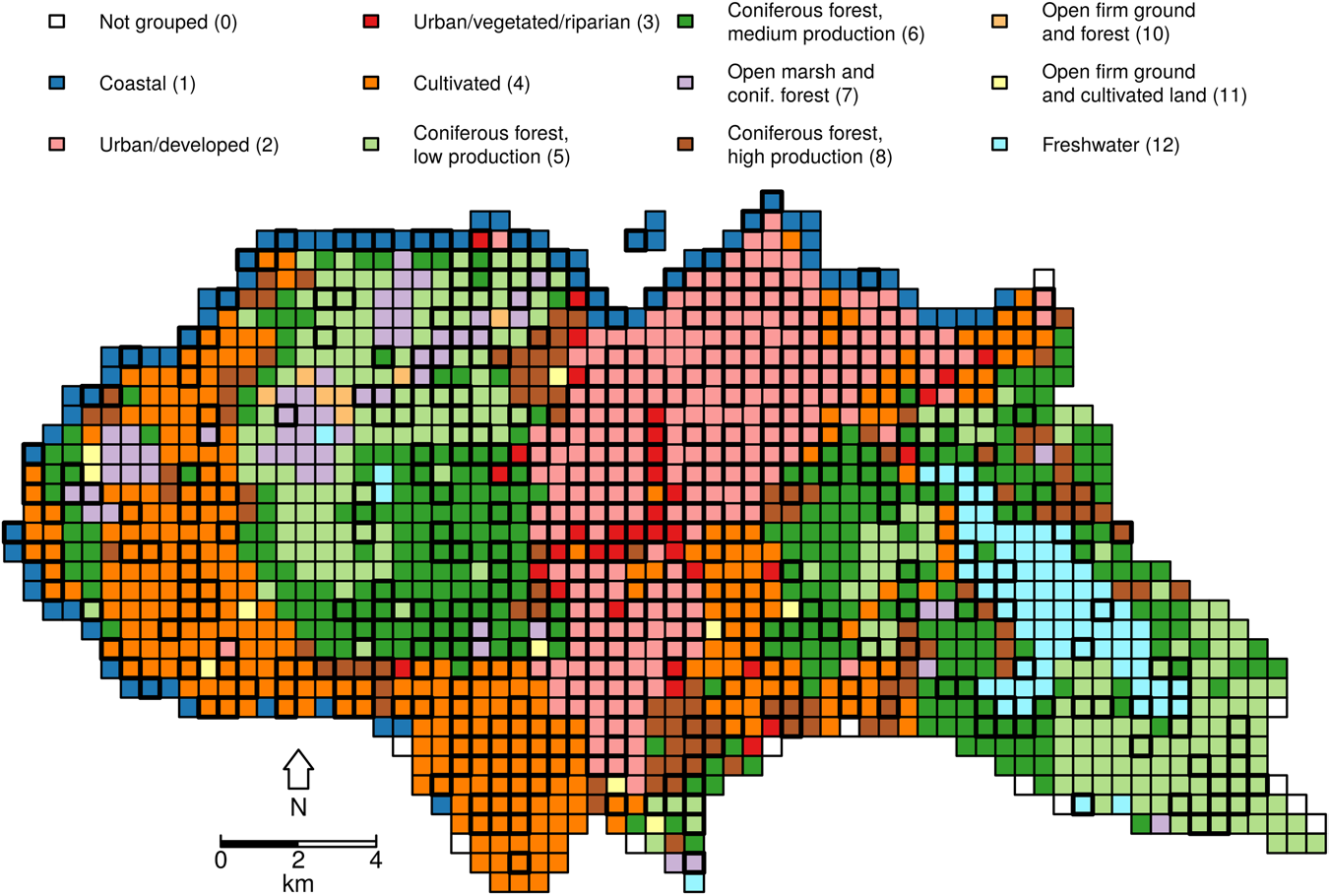
Trondheim Municipality coloured by habitat. Colour definitions shown in the legend. Numbers and names refer to cluster number and the given habitat name. Grid cells used for modelling are indicated with a black border. The figure was made in R, version 3.6.1^55^.

The habitats were named according to the (on average) dominating land cover types within the cells (Fig. 2, Supplementary Material 1, Fig. S.1). The number of grid cells per habitat was median 76.5 (interquartile range 30-242.25, Table 1). The most frequent habitat within the municipality was *Cultivated*, followed by *Coniferous forest* and *Urban/developed areas*.

**Table 1:** Distribution of grid cells among habitats.

Habitat no.	Name	No. (total)	No. (models)
0	Not grouped	12	0
1	Coastal	79	26
2	Urban/developed	249	142
3	Urban/vegetated/riparian	36	15
4	Cultivated	319	122
5	Coniferous forest, low production	240	51
6	Coniferous forest, medium production	315	68
7	Open marsh and coniferous forest	59	15
8	Coniferous forest, high production	109	28
10	Open firm ground and forest	7	0
11	Open firm ground and cultivated land	10	0
12	Freshwater	74	18
	*Sum*	1509	485

The grid cells in the Not grouped-habitat include six clusters containing ≤ 3 grid cells. The number of grid cells used for modelling were the ones fulfilling the criteria listed in the methods. All grid cells were used for the predictions, except for habitat 10 and 11, as no grid cells from these habitats were included in the model building, thus having values undefined for the parameter.

Outliers in the number of records or number of species (evaluated separately for each taxon level) were excluded based on Tukey’s method (0.75 quantile + 1.5*IQR). Subsequently, the habitats *Open firm ground and forest* and *Open firm ground and cultivated land* were excluded from the analyses, as only one and two grid cells remained, respectively. 485 grid cells were included in the subsequent analyses (Fig. 2).

Habitat heterogeneity was calculated for each grid cell as the Simpson’s Diversity Index. The index is calculated as 1-*D*, where *D* is $D={\sum }^{}{\left(\frac{n}{N}\right)}^{2}$, and *n* is the total area of a particular land cover type, *N* is the total area of the grid cell. The index ranges between zero (completely homogeneous land cover) and one (infinite heterogeneity in land cover; a hypothetical value). The grid cells included in the analyses ranged between 0.012 and 0.884.

### Statistical analyses

Generalised Linear Mixed Effects Models (GLMM) were constructed, predicting the threatened- (Poisson error distribution), alien- (Poisson error distribution), and overall (negative binomial error distribution due to overdispersion) number of species in each grid cell by habitat, habitat heterogeneity, northness, including an interaction with taxon (birds, non-avian animals, plants, fungi and other taxa) for all variables. Total number of records within grid cells were used as offset to account for differences in sampling effort. To account for spatial autocorrelation (*Moran’s I* > 1 in exploratory Generalised Linear Models), a Matérn correlation function was used as a random effect (package spaMM^54^). The models were fitted using Maximum Likelihood. Model selection was performed as stepwise backwards selection, based on AIC on the full models of the form: $No.\,species=habitat\ast taxon+habitat\,heterogeneity\ast taxon+northness\ast taxon+\,Matern(1\,|\,longitude+latitude)$. The models were subsequently used to predict species richness across all grid cells within the Trondheim municipality, using 100 records as an offset.

All data preparation, analyses and figures were made in R, version 3.6.1^55^.

## Results

**Figure 3:**
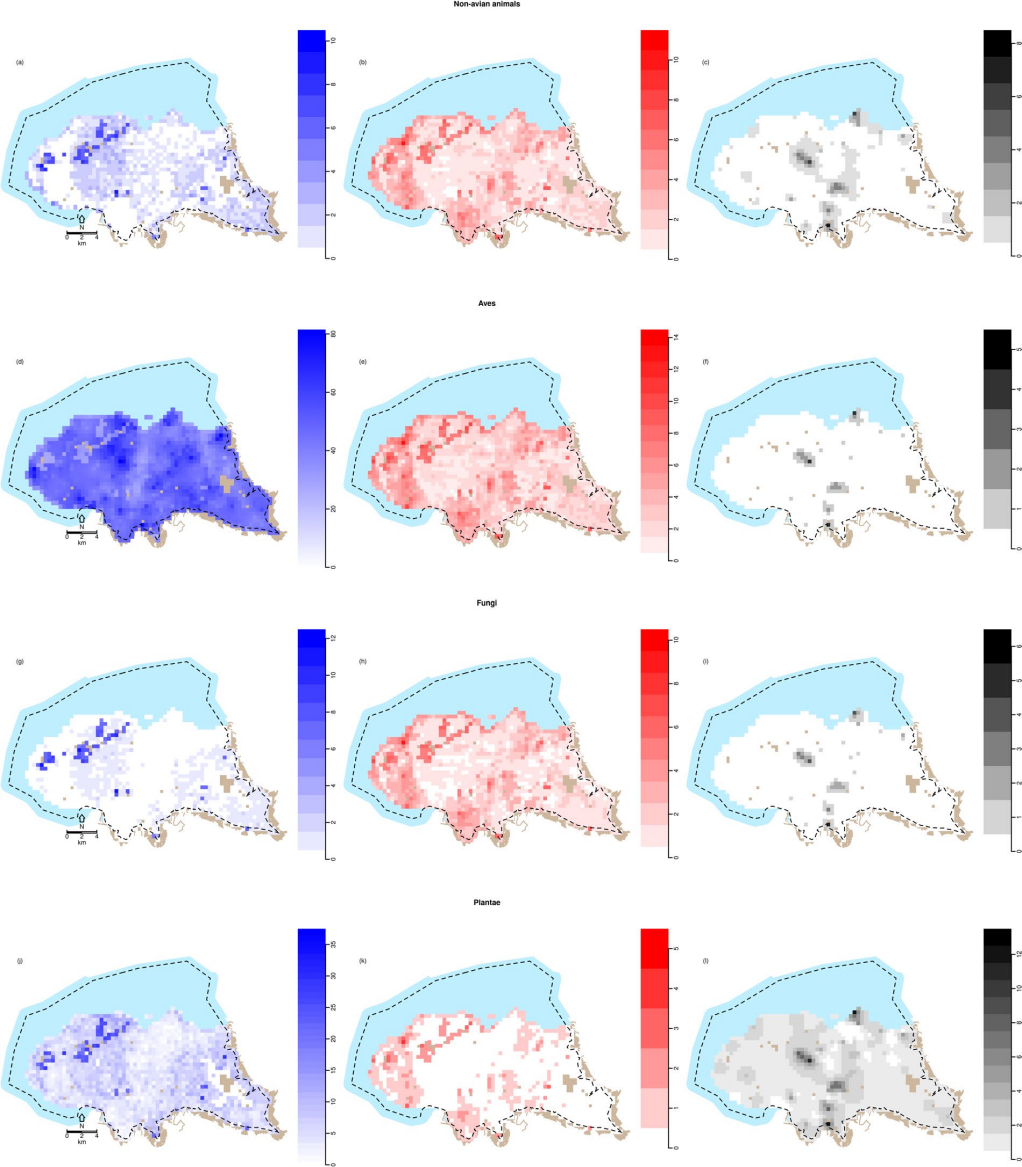
Maps of predicted species richness. Predicted number of species for each taxonomic- and species group given the realised habitat, habitat heterogeneity and northness. All predictions were made using 100 records (i.e. sampling events) as the offset. (**a**) Non-avian animals in total; (**b**) Threatened non-avian animals; (**c**) Alien non-avian animals; (**d**) Birds in total; (**e**) Threatened birds; (**f**) Alien birds; (**g**) Fungi in total; (**h**) Threatened fungi; (**i**) Alien fungi; (**j**) Plants in total; (**k**) Threatened plants; (**l**) Alien plants. The figure was made in R, version 3.6.1^55^.

**Figure 4:**
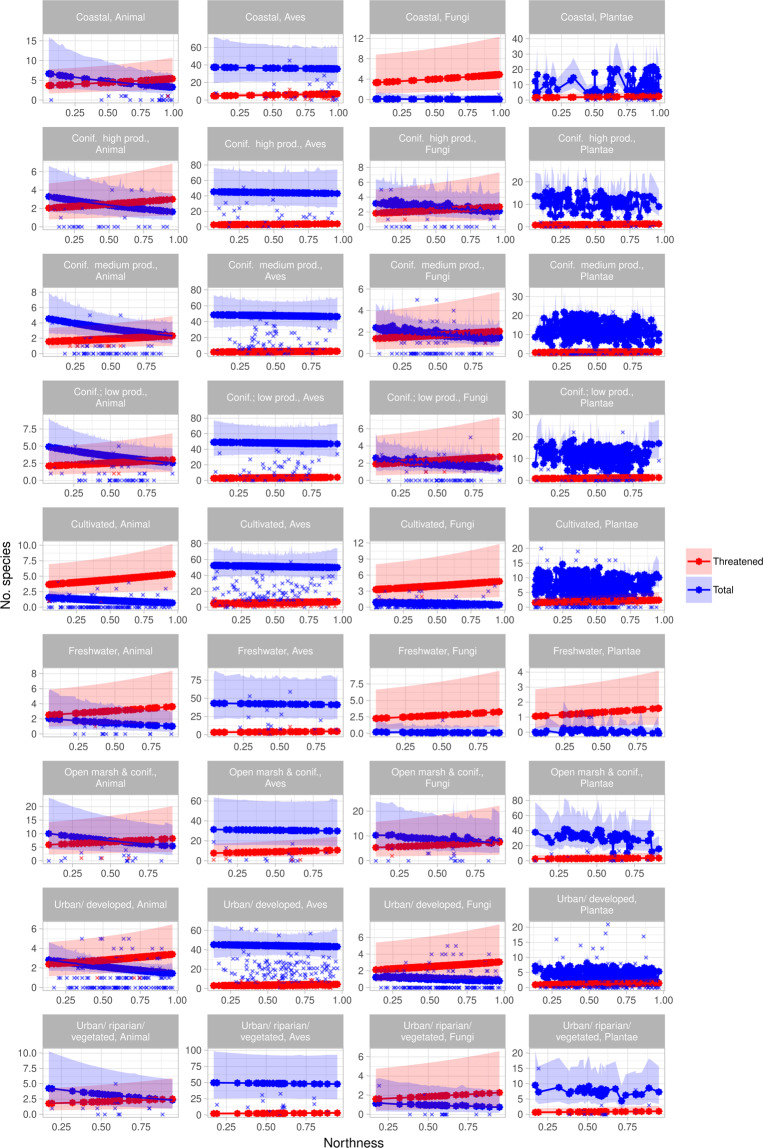
Effect of northness. Effect of northness on predicted species richness across taxa and within habitats. Crosses indicate observed values (incl. spatial effects and variations in all predictors), filled circles are the predictions (spatial effects removed, and all other predictors set to their mean values), and ribbons indicate 0.95 confidence intervals around the predictions. Note the different y-axes. The figure was made in R, version 3.6.1^55^.

**Figure 5:**
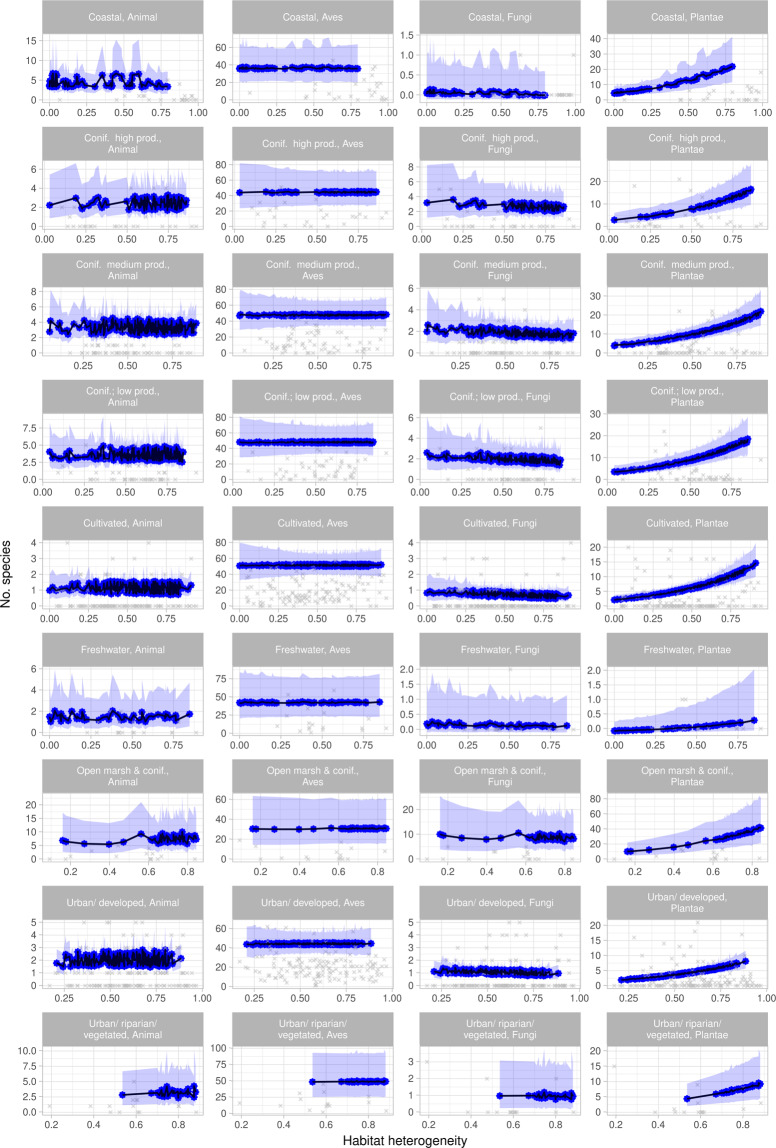
Effect of habitat heterogeneity. Effect of habitat heterogeneity on predicted species richness across taxa and within habitats. Crosses indicate observed values (incl. spatial effects and variations in all predictors), filled circles are the predictions (spatial effects removed, and all other predictors set to their mean values), and ribbons indicate 0.95 confidence intervals around the predictions. Note the different y-axes. The figure was made in R, version 3.6.1^55^.

**Figure 6:**
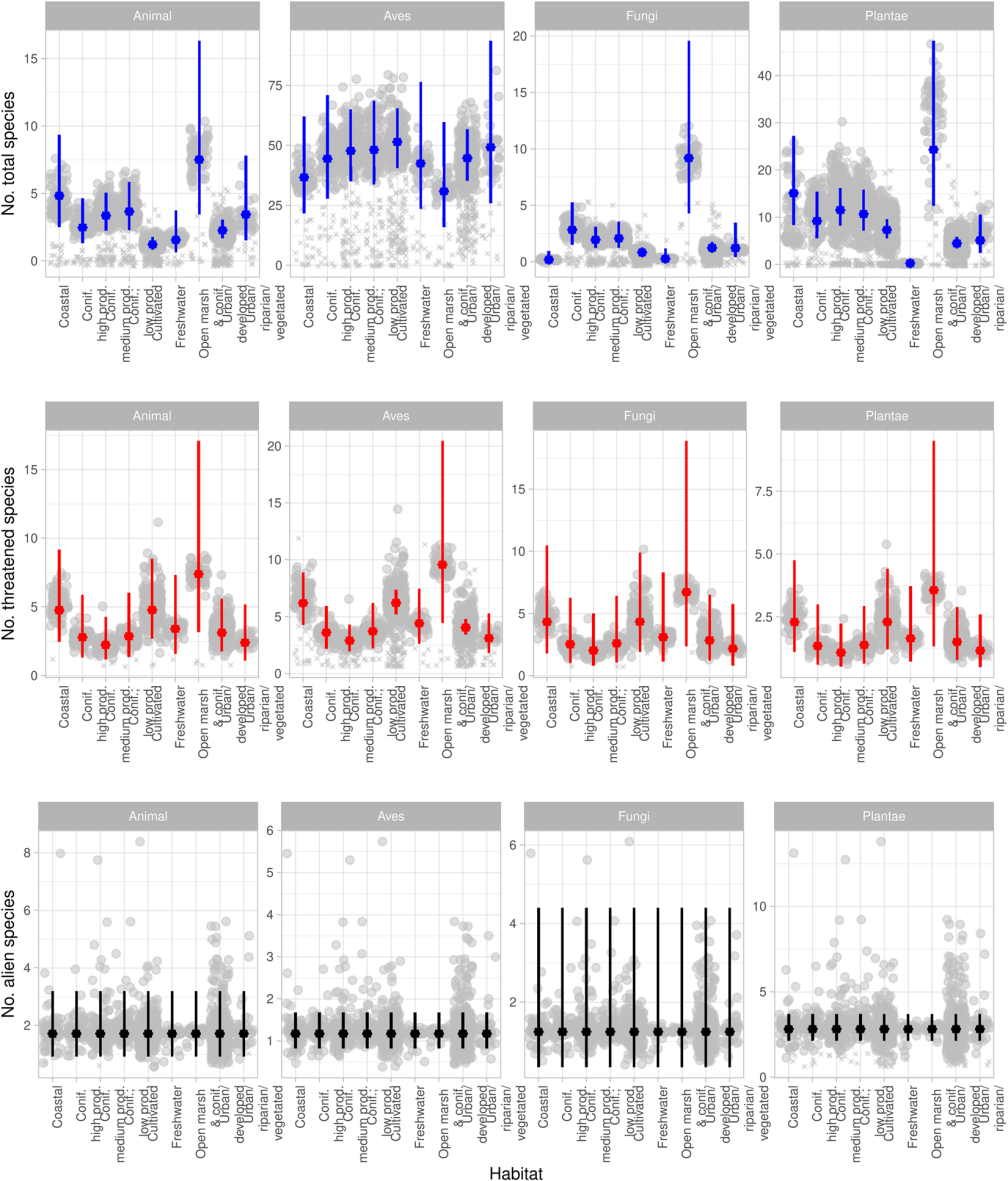
Effects of habitat and taxonomic group. Effect of habitat on predicted species richness across taxa and within habitats. Crosses indicate observed values (incl. spatial effects and variations in all predictors), filled grey circles are the predictions (incl. spatial effects and variations in all predictors), coloured circles indicate mean predicted values (spatial effects removed, and all other predictors set to their mean values), and lines indicate the 0.95 confidence interval of the prediction. Note the different y-axes. The figure was made in R, version 3.6.1^55^.

Different models proved to be optimal for the three species groups (all-, threatened- and alien species). For overall species richness, all predictors and interaction terms were retained, whereas threatened species richness was predicted by habitat, northness and taxon. Alien species richness was only predicted by taxon (Tables 2–4, Figs. 3–6). The spatial correlation parameters for total- (ν=0.460, ρ=0.001), threatened- (ν=2.254, ρ=0.005), and alien (ν=0.759, ρ=0.002) species richness indicate that all model predictions are spatially correlated (Tables 2–4). Plotting the estimated correlations against distances indicate total species richness having the farthest-reaching correlations, followed by alien and threatened species richness (Supplementary material 4, Fig. S.2).

**Table 2:** Model output, total species richness.

*Fixed effects (family: negative binomial, shape = 0.789)*					
Marginal AIC: 8014.325			Estimate	Cond.SE	t-value
(Intercept)			−0.979	0.347	−2.819
Urban/developed			0.198	0.288	0.689
Urban/vegetated/riparian			0.295	0.428	0.689
Cultivated			0.339	0.297	1.142
Coniferous forest, low production			0.273	0.323	0.845
Coniferous forest, medium production			0.265	0.314	0.841
Open marsh and coniferous forest			−0.172	0.433	−0.396
Coniferous forest, high production			0.192	0.367	0.525
Freshwater			0.148	0.399	0.371
Plantae			−1.998	0.503	−3.975
Animal			−1.660	0.545	−3.047
Fungi			−4.834	0.938	−5.156
Habitat heterogeneity			0.007	0.326	0.021
Northness			−0.056	0.287	−0.194
Urban/developed: Plantae			−1.418	0.402	−3.525
Urban/vegetated/riparian: Plantae			−1.378	0.612	−2.253
Cultivated: Plantae			−1.063	0.421	−2.526
Coniferous forest, low production: Plantae			−0.621	0.458	−1.355
Coniferous forest, medium production: Plantae			−0.534	0.443	−1.205
Open marsh and coniferous forest: Plantae			0.648	0.599	1.082
Coniferous forest, high production: Plantae			−0.685	0.521	−1.314
Freshwater: Plantae			−4.267	0.930	−4.589
Urban/developed: Animal			−0.956	0.434	−2.206
Urban/vegetated/riparian: Animal			−0.634	0.663	−0.956
Cultivated: Animal			−1.719	0.468	−3.677
Coniferous forest, low production: Animal			−0.551	0.501	−1.099
Coniferous forest, medium production: Animal			−0.628	0.485	−1.294
Open marsh and coniferous forest: Animal			0.612	0.654	0.935
Coniferous forest, high production: Animal			−0.860	0.581	−1.480
Freshwater: Animal			−1.281	0.655	−1.954
Urban/developed: Fungi			1.684	0.862	1.952
Urban/vegetated/riparian: Fungi			1.569	1.052	1.492
Cultivated: Fungi			1.142	0.883	1.294
Coniferous forest, low production: Fungi			2.126	0.906	2.345
Coniferous forest, medium production: Fungi			2.071	0.894	2.316
Open marsh and coniferous forest: Fungi			4.054	0.988	4.103
Coniferous forest, high production: Fungi			2.513	0.945	2.659
Freshwater: Fungi			0.215	1.160	0.185
Plantae: Habitat heterogeneity			2.002	0.481	4.165
Animal: Habitat heterogeneity			−0.022	0.528	−0.041
Fungi: Habitat heterogeneity			−0.367	0.604	−0.608
Plantae: Northness			−0.084	0.398	−0.211
Animal: Northness			−0.681	0.453	−1.503
Fungi: Northness			−0.423	0.501	−0.844
***Random effects (family: Gaussian)***					
**Correlation parameters**		**Variance parameters**			
**ν**	**ρ**	**λ**			
0.460	0.00123	0.118			

Model output from the spatially correlated GLMM of total species richness. The model was constructed with a negative binomial error structure. The factor levels *Coastal* and *Aves* are used as intercepts, thus categorical predictor values are relative to these.

**Table 3:** Model output from the spatially correlated GLMM of threatened species richness.

*Fixed effects (family: Poisson)*			
Marginal AIC: 1400.967	Estimate	Cond.SE	t-value
(Intercept)	−2.982	0.248	−12.022
Urban/developed	−0.420	0.195	−2.151
Urban/vegetated/riparian	−0.681	0.322	−2.114
Cultivated	0.003	0.204	0.015
Coniferous forest, low production	−0.506	0.314	−1.611
Coniferous forest, medium production	−0.753	0.272	−2.764
Open marsh and coniferous forest	0.436	0.431	1.013
Coniferous forest, high production	−0.533	0.311	−1.714
Freshwater	−0.333	0.325	−1.026
Plantae	−0.987	0.320	−3.089
Animal	−0.259	0.283	−0.912
Fungi	−0.353	0.411	−0.858
Northness	0.384	0.234	1.642
***Random effects (family: Gaussian)***			
**Correlation parameters**		**Variance parameters**	
**ν**	**ρ**	**λ**	
2.254	0.00511	0.1984	

The model was constructed with a Poisson error structure. The factor levels *Coastal* and *Aves* are used as intercepts, thus categorical predictor values are relative to these.

**Table 4:** Model output from the spatially correlated GLMM of alien species richness.

*Fixed effects (family: Poisson)*			
Marginal AIC: 712.727	Estimate	Cond.SE	t-value
(Intercept)	−4.441	0.80	−24.715
Plantae	0.878	0.167	5.254
Animal	0.390	0.327	1.164
Fungi	0.059	0.645	0.092
***Random effects (family: Gaussian)***			
**Correlation parameters**		**Variance parameters**	
**ν**	**ρ**	**λ**	
0.759	0.00178	0.597	

The model was constructed with a Poisson error structure. The factor level *Aves* is used as intercept, thus categorical predictor values are relative to this.

### Total species richness

For overall species richness, northness had a negative effect on species richness, the magnitude varying with taxon (Table 2, Fig. 4): non-avian animals responded most strongly to northness, followed by fungi, plants and birds. The response to habitat heterogeneity varied by taxon: plants and birds responding positively to increasing levels of habitat heterogeneity, fungi and non-avian animals having a negative response (Fig. 5). Similarly, the response to habitat differed among taxa, all other variables being held constant at mean values: fewest birds are predicted in *Open marsh and coniferous forest* followed by *Coastal*, *Freshwater, Coniferous forest; high production* and *Urban/developed*. The highest number was predicted for *Cultivated*, followed by *Urban/vegetated/riparian, Coniferous forest; low production* and *–medium production*. However, 0.95 C.I. overlapped for all habitats. For non-avian animals, *Cultivated* and *Urban/developed* had lower predicted species richness compared to *Open marsh and coniferous forest*, and *Cultivated* was lower than *Coniferous forest; low production* and *–medium production* as well. All other 0.95 C.I. overlapped. The highest number of fungi species was predicted for *Open marsh and coniferous forest*, 0.95 C.I. only overlapping with *Coniferous forest; high production*. The lowest number was predicted for *Coastal*, 0.95. C.I. overlapping with *Freshwater*, *Cultivated*, *Urban/vegetated/riparian* and *Urban/developed*. The lowest number of plants was predicted for *Freshwater*, followed by *Urban/developed* (0.95 overlapping with *Urban/vegetated/riparian*, *Cultivated* and *Coniferous forest; high production*). The highest number was predicted for *Open marsh and coniferous forest*, 0.95 C.I. overlapping with *Coastal*, *Coniferous forest; medium production*, *- low production* and *Coniferous forest; high production* (Fig. 6).

### Threatened species richness

For threatened species, increasing values of northness increase the predicted number of species (Table 3, Fig. 4). The highest species richness values are found for birds, followed by non-avian animals, fungi and plants. However, 0.95 C.I. overlap for all taxa except for birds and plants in *Urban/developed-* and *Cultivated* areas. The highest numbers of species are found in *Open marsh and coniferous forest*, followed by *Cultivated*, *Coastal, Freshwater, Urban/developed, Coniferous forest; low production*, - *high production, Urban/vegetated/riparian*, and *Coniferous forest*; *medium production*. However, all 0.95 C.I. overlap (Fig. 6).

### Alien species richness

For alien species, only taxon was retained as a predictor; the highest number of species predicted for plants, followed by non-avian animals, fungi and birds. However, the 0.95 C.I. overlapped for all taxa except birds and plants (Table 4, Fig. 6).

## Discussion

Urban areas are often found to have high levels of biodiversity, but little is known on how fine-scale land use is structuring species diversity in cities. We used species occurrence records from GBIF and official land cover classifications to determine how habitat affects total species richness, and the number of threatened and alien species. We did so by constructing spatially correlated Generalised Linear Mixed Effects Models based on habitat, habitat heterogeneity, aspect and taxonomic group within 500 m × 500 m grid cells across the Trondheim municipality, selecting the best models based on ∆AIC. The best models varied for overall-, threatened and alien species richness, with total species richness depending on all predictors and their interaction with taxon, whereas threatened species richness depended on habitat, aspect and taxon, and alien species richness only depended on taxon. The relationship between species richness in general are highly complex and dependent on multiple factors and interactions (Table 2, Figs. 3–6). Threatened, native species are associated with non-anthropogenic habitats (Table 3, Figs. 4 and 6), whereas alien species are mainly affected by spatial correlations on the investigated spatial scale (Table 4). The key findings of this study advance our understanding of the field by confirming the association of threatened, native species with more natural habitats, and the potential for establishment of alien species across all habitats on a management-relevant spatial scale.

The retention of all predictors and interactions in the model of overall species richness illustrate the complex relationships between environmental variables and different taxonomic groups. Nevertheless, the overall negative effect of northness reflect the low species richness of north-facing slopes, compared to south-facing ones^42^ (Fig. 4). The different taxa responded differently to increasing habitat heterogeneity, the only unidirectional response being for plants (positive) (Fig. 5). This supports the results of Matthies *et al*. (2017)^30^ and Beninde *et al*.(2015)^56^, in which respectively habitat heterogeneity and habitat richness were positively associated with species richness in urban areas. However, other studies have found a positive correlation for restricted taxonomic groups, such as arthropods^29,40^, birds and mammals^30^, which was not observed here.

Non-surprisingly, the different taxa responded differently to various habitats (Fig. 6). Interestingly, whereas plants, fungi and non-avian animals generally responded negatively to urban areas (differences not necessarily significantly different from other habitats however), the effect was less pronounced for birds. This could reflect their high mobility, and potential for an “urban adapter/exploiter”-status of some bird species^13^. In contrast, the habitat with the highest predicted number of both plant-, non-avian animal-, and fungi species, had the lowest predicted number of bird species.

Threatened species richness generally responded positively to increasing northness, in contrast to what would be expected (Fig. 4). This could potentially be an artefact of the habitat associations; *Coastal* areas had higher northness values (*mean* = *0.758, S.E.* = *0.022* compared to the overall *mean* = *5.35, S.E.* = *0.005*). The effect of taxon reflects the differences in the number of species within each taxonomic group being classified as threatened; (50 bird species, 26 plant species, 12 non-avian animal species, 33 fungi species included in the study). For all taxa, the lowest species numbers are predicted for all variants of coniferous forest, contrary to the initial expectations, and urban areas (Fig. 6). The negative effect of urban areas on threatened species richness mirrors the findings of Aronson *et al*. (2014)^8^, and emphasises how vulnerable native species are not pre-adapted to the changed environments of the city. Contrary to expectations, the effect of the various forest habitats on threatened species is lower than for most other habitats (Table 3). The low number of threatened species in forests can be due to the lack of sampling, showing a spatial bias in the data rather than an effect. This should however be accounted for by using the number of records as an offset in the models. Rather, large parts of the forested areas in Trondheim are srongly affected by previous afforestation for timber production, where mainly coniferous species (both native and alien) were planted^33^. These forests might not provide the needed conditions for native species^57^. Plantations and secondary vegetation have been shown to harbour fewer species than primary forests^58,59^. The lack of association between forested areas and threatened species calls for a nuanced perspective on what forest types constitute suitable habitats for species of interest, as indicated by Ingram *et al*. (2015)^58^ and Horák *et al*. (2019)^59^. The highest species numbers are predicted for *Open marsh and coniferous forest* and *Coastal* areas (Fig. 6); the former is likely the habitat category reflecting the lowest human impact. The high number of threatened species in coastal habitats can likely be ascribed to these habitats being ecotones, providing varied habitat conditions. Ecotones have been suggested to have an increased species richness^36^. Lloyd *et al*. (2000)^38^ found ecotonal species to mainly be natives, which is supported by the findings here.

Interestingly, in the model of alien species richness, only taxon was retained as a significant predictor, reflecting the differences in the number of species within each taxonomic group being classified as alien (5 bird species, 156 plant species, 10 non-avian animal species, 6 fungi species included in the study). The lack of response to either of the other investigated variables stands in stark contrast to the expectations and previous findings, but can be attributed to alien species often being generalist opportunists; the spatial scale investigated does not reflect the fine-scale conditions affecting the individual species. This result highlights that on this spatial scale, all parts of the municipality are open for potential invasion by alien species. Given the spatial correlations (Supplementary material 4, Fig. S.2), it is evident that founder events and subsequent spread of alien species are of crucial importance: on the investigated scale, even more important than the configuration of environment. As many alien species are introduced through urban areas mainly due trade and traffic^12,15,31^, emphasis must be put on the importance of avoiding unintentional introduction of potential invasive species. As an example, the review by Kowarik (2011)^5^ found cities to be hotspots of alien plant species. In addition, port cities have been suggested as even greater hotspots of introductions, leaving Trondheim even more vulnerable^60,61^.

As the explanatory variables used in these models are “indirect” (*sensu* Guisan and Zimmermann (2000)^62^), the habitats are proxies for underlying environmental (direct) drivers. Therefore, a direct extrapolation to other geographical areas should be cautious^62^. However, the general methods are highly applicable elsewhere.

Of the 1,509 grid terrestrial cells, 485 qualified for analyses; species occurrence data was sparse in the rest. Those used in the analyses were biased towards urban areas (Table 1), supporting the general trend in citizen science data; concentrated around inhabited or areas otherwise accessible to the public^63,64^. For example, areas within Trondheim municipality relatively far from human activity are under-sampled, with two habitats not being represented in the analyses at all (*Open firm ground and forest –and cultivated land*). This bias was accounted for in the models, but the differentiated sampling effort nevertheless leaves varying degrees of uncertainty for each habitat and taxon. The sample sizes differed among species groups, with many more observations of threatened than alien species. The differences thus might reflect sampling strategy rather than reality.

As the models are by nature rather crude, they inevitably lack predictor variables, which could have increased model accuracy. However, including highly detailed variables was not the aim of this study. Since the data set included a wide array of species, these will not respond in similar ways to variation in the included variables, or to missing variables^65^. The more species included in the models, the more opposing mechanisms are attempted to be fitted within a single modelling framework, giving a poorer fit, compared to models with a narrower scope.

The number of GBIF records have increased in recent years (see Speed *et al*. (2018)^64^). Of all species recorded in Trondheim, approximately 1/3 have been recorded within the municipality from year 2013 to 2018. Of the 6,020 species from the downloaded data set not included in the analyses, 33.9% (2,039) have only been recorded once, and 85.5% (5,150) have been recorded <10 times. Most of these infrequent species are insects. This taxonomic skew is likely due to this species group being poorly sampled or requiring expert knowledge to identify to species level.

Different correlations with environmental variables are expected at different spatial scales for different organisms^19,23,66^. Taxa and species with opposing responses to the included variables could mask each other, thus not revealing the underlying mechanisms^56^. Simultaneously, the mechanisms underlying species distributions vary with spatial scale, not necessarily in the same direction for different taxa^19,23,67^. As multiple different taxonomic groups were included in this study, the used spatial scale is potentially inappropriate for all taxa.

According to Pautasso (2007)^19^, a negative correlation between urbanisation and species richness is expected when the study grain is smaller than 1 km, as in this study, but positive at larger scales. This is ascribed to the larger scale reflecting human settlement in productive areas, competing for space with other species, whereas the small scales reflect more detailed environmental- and land cover effects.

Our results indicate that if the Trondheim municipality is to be managed to favour biodiversity, favouring threatened species and excluding alien species, the following actions can be recommended:

Habitat heterogeneity on a relatively small spatial scale should be ensured, favouring overall species richness. This should however not be confused with fragmentation of natural habitat.

To favour threatened species, non-anthropogenic- and coastal areas should be monitored and protected, potentially expanding the actions to ecotones in general.

To limit the spread of alien species, initial introduction and establishment should be avoided. Thus, urban- and other anthropogenic areas should be closely monitored and managed^12,68^.

Protection of important and heterogeneous habitat should be accounted for in unison with ensuring large habitat patches, rather than multiple smaller ones; a metastudy by Beninde *et al*. (2015)^56^ showed patch area to have the largest positive effect on urban biodiversity.

## Conclusions

Overall-, threatened- and alien species richness are not determined by the same land-cover variables. Total- and threatened species richness responds to both habitat and aspect, whereas alien species richness does not respond to any of the variables included in this study. The highest numbers of threatened species are associated with non-anthropogenic habitats, but in contrast to expectations, not more positively associated with forested areas than other habitats, calling for detailed investigations of the importance of forest environments for threatened species. Our finding that alien species do not respond to land-cover variables, but only spatial correlations, confirms the importance of founder events, and highlights the status of cities as gateways for alien species in general.

To mitigate the knowledge gaps from under-sampled habitats, we urge for sampling outside inhabited areas and for less investigated taxa. Using models build on administrative land cover maps and open database occurrence records can be a useful tool for local biodiversity management, by providing guidelines regarding where to aim future efforts, both regarding future conservation efforts and future investigations. Further work is however needed in dealing with the inherent biases of such databases.

In the case of Trondheim, an averaged sized Northern European city, the recommendations for biodiversity management are to ensure protection of natural habitats within a spatial resolution of 250,000 m^2^, and to closely monitor and manage urban areas to mitigate the introduction and spread of alien species.

## Data availability

All relevant data is available from public repository (GBIF Occurrence Download – March 6^th^ 2018, 10.15468/dl.ruacxc).

## Supplementary information

Supporting information.
