# Supplementary material for: Urban aliens and threatened near-naturals: Land-cover affects the species richness of alien- and threatened species in an urban-rural setting
Source: Sci Rep. 2020 May 22;10:8513. doi: 10.1038/s41598-020-65459-2 (PMC7244569; doi:10.1038/s41598-020-65459-2)
Supplement: Supplementary file 1 — Supporting information. [file 41598_2020_65459_MOESM1_ESM.pdf]

Supplementary material

Urban aliens and threatened near-naturals: Land-cover affects the species richness of alien- and threatened species in an urban-rural setting

Tanja K. Petersen[1,2,*], James D.M. Speed[1], Vidar Grøtan[2] & Gunnar Austrheim[1]

[1] Department of Natural History, NTNU University Museum, Norwegian University of Science and Technology (NTNU), Erling Skakkes gt 47b, Trondheim, Norway

[2] Centre for Biodiversity Dynamics, Department of Biology, NTNU, NO-7491 Trondheim, Norway

*** Corresponding author:** tanja.k.petersen@ntnu.no

ORCID: Tanja K. Petersen: https://orcid.org/0000-0002-7599-712X

James D.M. Speed: http://orcid.org/0000-0002-0633-5595

Supplementary material

1. Land cover characteristic of the habitats

The characteristic AR5 land cover of each habitat are evaluated based on the mean area of each land cover (m^2) within the grid cells assigned to the respective habitats. An overview of the characterising land covers can be seen in Fig. S1. The dominating land cover has been determining for the used name for each of the habitats.

The official AR5 land covers falling under the labels defined here are as shown in Table S.1 (Ahlstrøm, Bjørklund and Frydenlund, 2014)[1].

The cluster analysis was based on the land cover within the classified grid cell. The analysis was also done by incorporating the land cover within the focal grid cell and the eight first order neighbour-cells, to smooth out the categorisation. The overall patterns were similar to what was found for the ``strict'' analysis, however a large degree of the more fine scale variation in land cover between categories was lost. Thus, further analyses were performed using habitat categories based only on the focal grid cell.

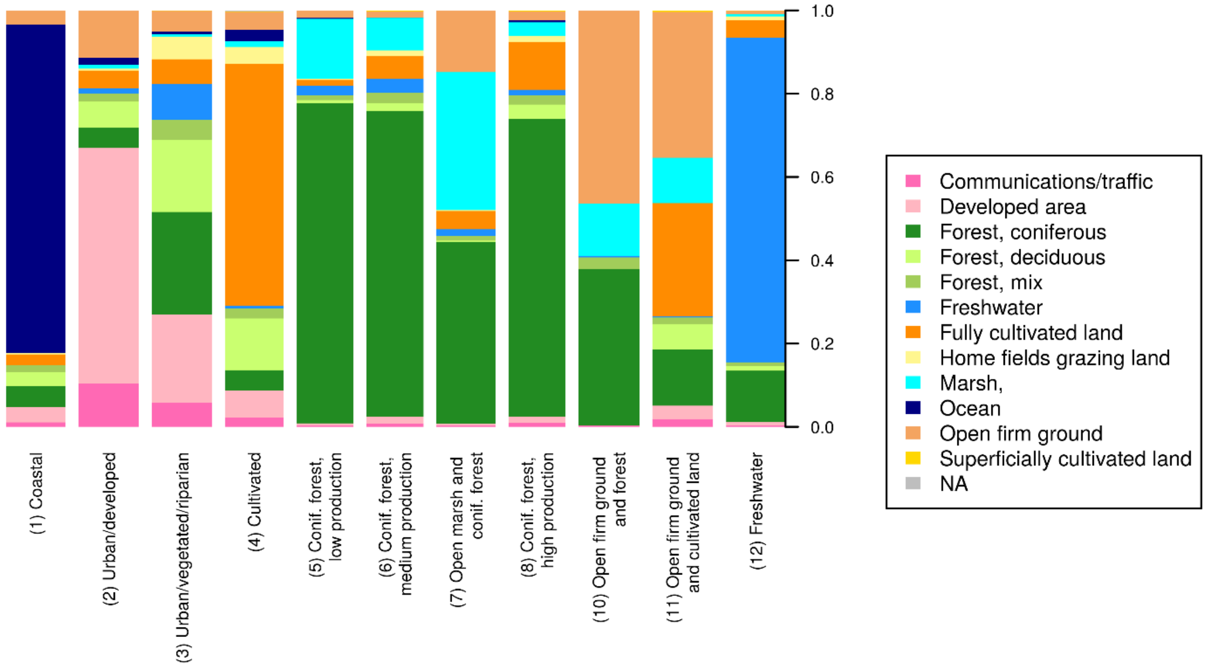

Figure S.1. Mean proportion of grid cell within each habitat covered by the respective AR5 land covers.

[1] Ahlstrøm, A. P., Bjørklund, K. and Frydenlund, J. (2014) *AR5 klassifikasjonssystem. Klassifikasjon av arealressurser*.

Supplementary material

Table S.1 Labels of AR5 land cover types in Fig. S.1 and the included sub-classes. Only sub-classes occurring within the study area are included. Categories are translated from Ahlstrøm et al. (2014).

Label	Area type	Tree cover	Productivity	Soil condition
Communications/traffic	Communications/traffic	-	-	-
Developed areas	Developed areas	-	-	-
Forest, coniferous	Forest	Coniferous	Impediment	Bedrock
				Shallow soil
				Soil
				Organic soil
			Low	Boulder
				Shallow soil
				Soil
				Organic soil
			Medium	Shallow soil
				Soil
				Organic soil
			High	Shallow soil
				Soil
				Organic soil
			Very high	Soil
Forest, deciduous	Forest	Deciduous	Impediment	Bedrock
				Shallow soil
				Soil
				Organic soil
			Low	Soil
				Organic soil
			Medium	Shallow soil
				Soil

				Organic soil
			High	Soil
				Organic soil
Forest, mix	Forest	Mixed	Impediment	Shallow soil
				Soil
				Organic soil
			Low	Shallow soil
				Soil
				Organic soil
			Medium	Shallow soil
				Soil
				Organic soil
			High	Soil
Freshwater	Freshwater	-	-	-
Fully cultivated land	Fully cultivated land	-	-	Soil
				Organic soil
Home fields grazing land	Home fields grazing land	Deciduous	-	Soil
		-	-	Shallow soil
				Soil
				Organic soil
Marsh	Marsh	Open	Impediment	-
		Coniferous	Impediment	-
			Low	-
			Medium	-
			High	-
		Deciduous	Impediment	-
			Medium	-
		Mix	Impediment	-
			Low	-

Ocean	Ocean	-	-	-
Open firm ground	Open firm ground	-	Impediment	Artificial surface
				Bedrock
				Boulder
				Shallow soil
				Soil
			Medium	Soil
			High	Soil
Superficially cultivated land	Superficially cultivated land	-	-	Shallow soil
				Soil
NA	-	-	-	-

Supplementary material

2. Distribution of data in taxonomic groups

Table S.2. Distribution of records between taxonomic groups. The distribution of the data from GBIF after the initial data cleaning procedure (described in the method section "*GBIF occurrence records*"), among taxonomic groups, including both number of records and number of registered species names. Capital letters indicate that subgroups are shown below in the table. Bold text indicate kingdom-level. "OTHER" indicate records/species not included in the animal-, plant- or fungi kingdom.

		**All species**	**Threatened species**	**Alien species**
**TOTAL**	Records	251,803	32,585	3,447
	Species	3,097	121	177
**ANIMALIA**	Records	239,038	32,351	2,226
	Species	1,353	62	15
Aves	Records	230,161	32,228	2,186
	Species	222	50	5
Mammalia	Records	729	97	7
	Species	28	4	1
Reptilia and Amphibia	Records	74	12	0
	Species	6	1	0
Arthropoda	Records	6,098	13	8
	Species	844	6	6
**PLANTAE**	Records	9,233	102	1,182
	Species	952	26	156
Bryophyta	Records	516	11	0
	Species	181	4	0
TRACHEOPHYTA	Records	8,481	91	1,182
	Species	702	22	156
Pinopsida	Records	357	0	124
	Species	23	0	15
Magnoliopida	Records	6,369	56	989
	Species	510	20	129
**FUNGI**	Records	3,369	132	39
	Species	763	33	6
**OTHER**	Records	163	-	-
	Species	29	-	-

3. Criteria for inclusion of threatened species

Description of the sorting of the Red List from the Norwegian Biodiversity Information Centre. The categories from 2006, 2010 and 2015 for each evaluated species evaluated were compared:

The official Norwegian Red Lists from 2006, 2010 and 2015, including the notes on the evaluations, were provided by the Norwegian Biodiversity Information Centre. The three lists provided the basis for the modified version of the Norwegian Red List used in this study. The used abbreviations are in line with the official IUCN categories: *DD* = Data Deficient, *LC* = Least Concern, *NT* = Near Threatened, *VU* = Vulnerable, *EN* = Endangered, *CR* = Critically Endangered, *RE* = Regionally Extinct, *NE* = Not Evaluated, *NA* = Not Available.

If species have not been evaluated in any year (categorised as either NA or NE), they are immediately discarded.

Similarly, if species were only evaluated as LC, or as any combination of LC and non-evaluated, they are discarded.

If species have previously been evaluated to DD (on the threatened part of the Red List), but was evaluated as LC in the latest version (2015), they were discarded.

Species listed as LC in the previous two assessments were discarded, regardless of their category in 2006.

All species evaluated to be in the threatened categories (RE, CR, EN, VU and NT) in 2015 were included in the used Red List. Species listed as DD were evaluated separately (see the further description).

All species always within the threatened categories were included in the list (incl. combinations with NA, NE and DD - thus, species never categorised as LC).

All species evaluated as data deficient in all years (DD) were included in the final version of the Red List. Similarly, all species listed as DD once, with any combination of NE and NA, were included.

All species listed as regionally extinct (RE) at any point, were included in the list.

For all species listed as LC, NA, NE or DD in the latest assessment (2015), but previously listed as any of the threatened categories (RE, CR, EN, VU, NT and DD), the notes on the reasoning for down-grading of the respective species were assessed and evaluated. Generally, species where there was great uncertainty regarding the actual current status of the species, was included in the list. Otherwise, the species was discarded.

Description/reasoning for all of the individually evaluated species can be presented upon requests.

4. Spatial correlation

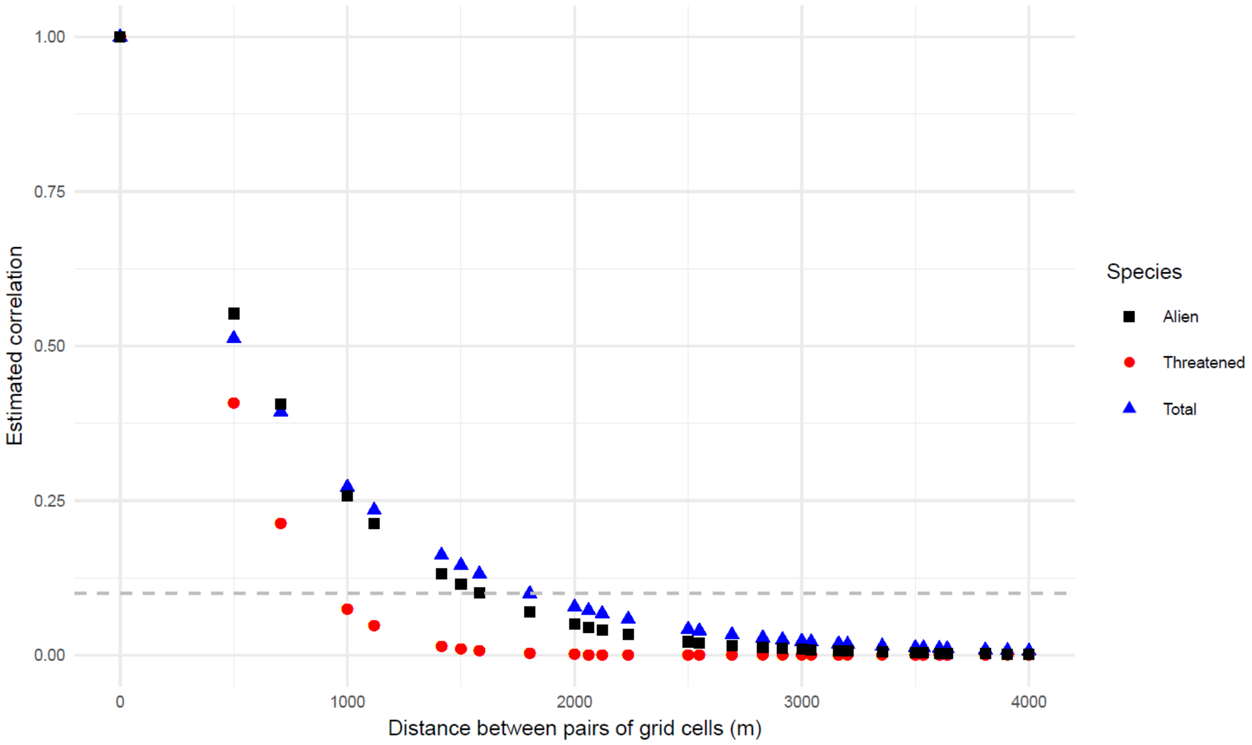

Figure S.2. Spatial correlation of models.

Estimated correlation of grid cells as a function of distance between the grid cells included in the models. Correlation- and distances are based on the spatial correlation parameters ν and ρ, as described in the model outputs (table 2-4, main text).
